# Alginate-Based Emulsions and Hydrogels for Extending the Shelf Life of Banana Fruit

**DOI:** 10.3390/gels10040245

**Published:** 2024-04-03

**Authors:** Silvio Iacovino, Martina Cofelice, Elena Sorrentino, Francesca Cuomo, Maria Cristina Messia, Francesco Lopez

**Affiliations:** 1 Department of Agricultural, Environmental and Food Sciences (DiAAA), University of Molise, Via De Sanctis, 86100 Campobasso, Italy; silvio.iacovino@unimol.it (S.I.); martina.cofelice@unimol.it (M.C.); sorrentino@unimol.it (E.S.); francesca.cuomo@unimol.it (F.C.); messia@unimol.it (M.C.M.); 2 Center for Colloid and Surface Science (CSGI), University of Molise, Via De Sanctis, 86100 Campobasso, Italy

**Keywords:** edible coating, sodium alginate, banana fruit, crosslinking agent

## Abstract

Edible coatings are used to extend the shelf life of various fruit, including bananas (*Musa* from the *Musaceae* family). After harvest, bananas reach the ripening and subsequent senescence phase. During senescence, the quality of the fruit deteriorates as it takes on a brown color and the tissue becomes soft. To extend the shelf life of such a fruit, effective methods to delay ripening are required. In this study, an alginate-based emulsion, i.e., an oil-in-water emulsion of lemongrass essential oil in alginate, was used to combine the mechanical properties of hydrocolloids with the water barrier properties of the oil phase. The emulsion was sprayed onto the whole fruit with an airbrush, and calcium chloride was added to promote gelling of the alginate. Compared to the uncoated fruit, coated bananas remained uniform in appearance (peel color) for longer, showed less weight loss, had a delay in the formation of total soluble solids, and in the consumption of organic acids. The shelf life of the coated fruit was extended by up to 11 days, at least 5 days more than uncoated bananas. Overall, the proposed coating could be suitable for reducing the global amount of food waste.

## 1. Introduction

Fruit and vegetables are an important part of our daily diet, as they contain vitamins and minerals that can contribute to maintaining good health. The choice of fruit and vegetables is largely influenced by parameters such as appearance, texture, and flavor, and they are best bought when they are in season. Otherwise, it is necessary to develop new strategies to ensure the proper freshness of this type of food. In general, climacteric fruits are metabolically active and perishable after harvest as they undergo a reduction in quality due to the natural ripening and senescence processes [1,2]. Controlling these processes is crucial for maintaining the overall quality and extending the shelf life of such products during storage, as their economic value depends on their freshness. Therefore, good management of the post-harvest phase can have a positive impact on the entire supply chain, from the farmer to the consumer. Edible coatings are a promising strategy to increase the longevity of fresh fruit and vegetables by reducing microbial growth, slowing down enzymatic browning, minimizing moisture loss, and thus ensuring lower post-harvest losses and better quality [3,4]. The use of edible coatings has been promoted as a safe, cost-effective, and easy way to extend the shelf life of fruit and reduce packaging waste [5,6]. The main components of edible coatings are hydrophilic food-grade biopolymers, which, when dispersed in water, form suspensions with the properties of colloids and are known as hydrocolloids. Hydrocolloids include carbohydrates and proteins with film-forming properties [4]. However, by combining the good mechanical properties of hydrocolloids with the water barrier properties of lipids, emulsion-based systems are being developed that can be used in various fields such as pharmaceutical, food, and biotechnology [7]. In general, the continuous phase of an oil-in-water emulsion is an aqueous solution of hydrophilic polymers (polysaccharides or proteins), while the dispersed phase consists of oil droplets. Considering the biopolymers, alginate isolated from bacteria and brown algae is a versatile, non-toxic, and inexpensive biopolymer with interesting technological properties [8,9,10]. Various studies have discussed the use of essential oils in the production of emulsion-based coatings, as they are natural compounds that are accepted by consumers and scientists mainly for their antimicrobial and antioxidant properties [11].

Among fruits, the banana (*Musa* from the *Musaceae* family) is one of the most consumed tropical products in the world market because it is cheap, nutritious, and flavorful. However, as a climacteric fruit, it has a relatively short post-harvest life [2], with an average global post-harvest loss of around 30% [12].

Various edible post-harvest coating formulations for bananas have already been tested, mainly using polysaccharides. As early as 2001, Kittur and colleagues [13] tested different coatings of chitosan, modified starch, and cellulose, mixed with glycerol monostearate to improve surface wettability, on mango and banana fruit. The results showed that the chitosan formulation led to a delay in weight loss, reduced the respiration rate, and protected against fungal infection. Chitosan was then combined with 1-methylcyclopropene, a synthetic compound commonly used to delay fruit ripening, for coating bananas, with the result showing that the peel aspect was kept uniform, and the commercial life of fruit was extended by 4 days [1]. Some years later, Gol and co-workers [14] followed the effects of different coatings applied on banana fruit for ten days and found that the combination of chitosan and gibberellic acid was effective in delaying weight loss, and the overall decay. In the same period, some Malaysian researchers used chitosan in combination with gum arabic to formulate a suitable edible coating to be applied by dipping in the bananas with the effect of extending their freshness during the storage period by 33 days [15], and more recently, a similar coating enriched with ZnO particles, with antibacterial properties, was applied on bananas and prolonged the shelf life of the fruit for more than 17 days of storage [16]. In 2018, Lustriane et al. [17], by monitoring starch content, weight loss, total soluble solids, pulp-to-peel ratio, and sensory quality found that chitosan in the form of nanoparticles could also extend shelf life of banana fruits, which was also confirmed by analyses of gene expression. Thakur and colleagues [18] also dealt with the formulation of edible coatings made of rice starch, carrageenan, and fatty acid ester of sucrose, and observed an improvement as well in the shelf life of the coated fruits. Recently, Deng et al. [19] used a coating realized by crosslinking alginate with whey protein isolate and followed the ripening process of bananas for 8 days, observing in this time window a meaningful difference between the coated and uncoated fruit.

There are few studies on the application of alginate-based edible coatings combined with essential oils for the preservation of bananas, such as those of Yang et al. [20] and Han et al. [21], who prepared sodium alginate/tea tree essential oil emulsions with TiO_2_ nanoparticles, and sodium alginate/carboxymethylcellulose films with cinnamon essential oil, respectively. Regarding the first study, although the authors observed the control of banana ripening, the European Food Safety Authority (EFSA) announced on 6 May 2021 that titanium dioxide can no longer be considered safe for use as a food additive due to its genotoxicity [22]. In a study by Han and colleagues [21], browning of the banana surface was observed within 7 days, despite the coating. In view of the literature presented, the aim of this study is to evaluate the effects of a food-grade emulsion-based edible coating crosslinked or not with sodium alginate and lemongrass essential oil on the physical and biochemical changes of banana fruit during storage. For this purpose, the coating formulations (alginate-based emulsion with lemongrass essential oil as dispersed phase, crosslinked with CaCl_2_ or not) were previously characterized in terms of their mechanical properties. The effect of their application on the fruit peel during ripening was then evaluated based on the development of various parameters, such as color, titratable acidity, total soluble solids, firmness, and sensory acceptability.

## 2. Results and Discussion

### 2.1. Characterization of Coating Formulations

**Figure 1:**
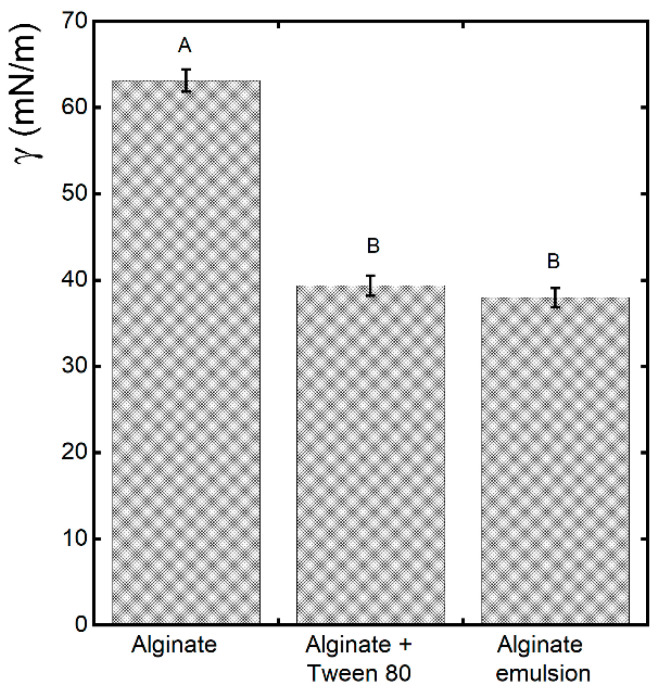
Mean values of surface tension (γ) of alginate, alginate and Tween 80, and alginate/LEO emulsion. Different letters(A and B) on column indicate significant difference among samples (*p*-value < 0.05).

The alginate-based emulsion was applied on banana peel through an airbrush and successful deposition was ensured by the decrease in surface tension associated with the formulation of the emulsion. Understanding surface tension is critical to the development of coatings that adhere well to food surfaces, have a uniform thickness, and provide a protective barrier against environmental influences [23]. Figure 1 shows the mean values of the surface tension of a suspension with 1% sodium alginate, the same suspension with 1% Tween 80, and the emulsion with 0.1% lemongrass essential oil (LEO). It can be observed that the use of Tween 80 was already effective in reducing the surface tension, while the presence of LEO in the formulation did not further reduce this parameter.

**Figure 2:**
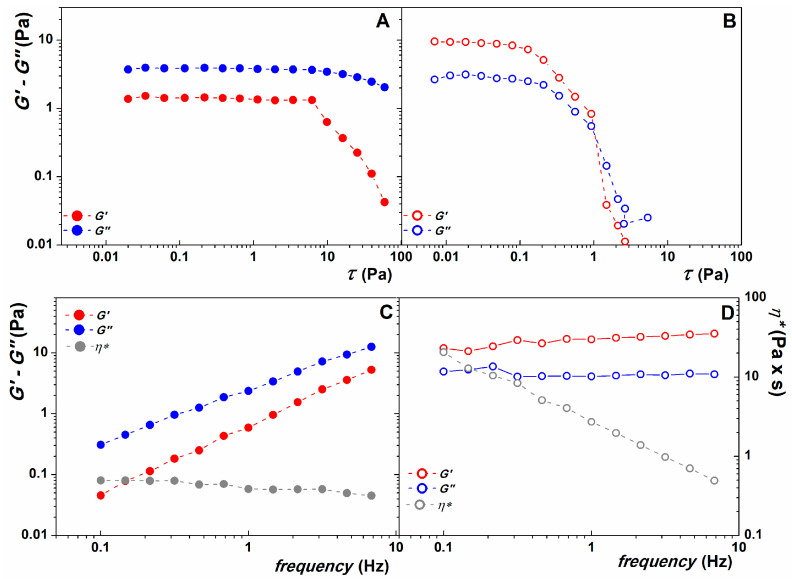
Amplitude (G′ and G″ as a function of shear stress, *τ*) and frequency sweep (G′ and G″ as a function of frequency) test made on alginate emulsion ((**A**) and (**C**), respectively) and on crosslinked hydrogel ((**B**) and (**D**), respectively). Complex viscosity, η*, is reported on right axes of panels (**C**) (for alginate emulsion) and (**D**) (for cross-linked hydrogel).

To understand the effects of adding CaCl_2_ as a crosslinking agent to the alginate emulsion, the mechanical properties of the coating film were analyzed before and after the application of CaCl_2_. Figure 2 shows the results of the rheological characterization. From the comparison of Figure 2A,B, in the amplitude sweep test, G″ prevailed over G′ in the alginate emulsion, indicating a liquid-like behavior consistent with the use of spray technology for application on the fruit surface, while after the application of the crosslinking agent, G′ was higher than G″, indicating the predominance of an elastic behavior. Moreover, depending on the material, the LVE range was longer for the alginate emulsion than for the crosslinked emulsion. Figure 2C,D shows the difference in the frequency dependence of the two systems. The stability of the crosslinked emulsion is stronger (no dependence on frequency in the applied range), which is typical for a hydrogel-like system. The alginate emulsion, on the other hand, shows a dependence on the applied frequency. Also, considering the complex viscosity η*, it is evident that the hydrogel was much more viscous than the non-crosslinked emulsion.

The preliminary characterization of the coating-forming formulations showed that the emulsion had suitable properties to be applied using the spray technique (liquid-like behavior) and to adhere to the fruit surface (low surface tension values), and that the application of the crosslinking agent promotes the formation of a hydrogel on the banana surface with elastic behavior.

### 2.2. Effect of Coating Application on Fruit Ripening

**Figure 3:**
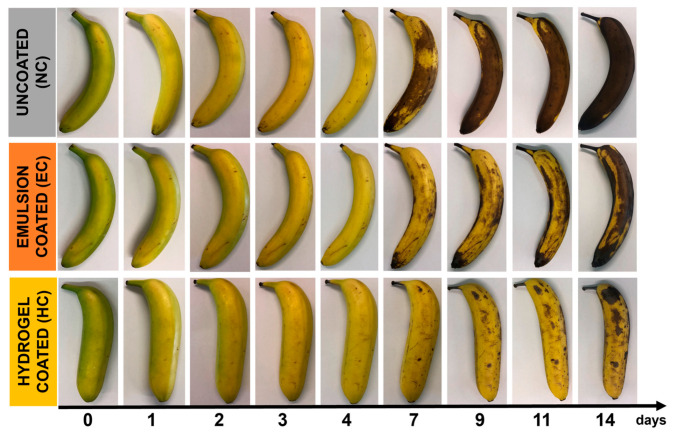
Appearance of coated and uncoated fruit as a function of the storage time.

Samples coated with alginate emulsion only (referred to as EC) with alginate crosslinked with CaCl_2_ forming hydrogel (HC) were stored at 20 °C together with the uncoated bananas (NC). The visual aspect of coated (EC and HC) and uncoated (NC) bananas during the storage is shown in Figure 3.

During ripening, the color of the banana peel color changes from green to brown and dark brown due to a series of enzymatic reactions. At the beginning of this period, chlorophyll is gradually converted into xanthophyll by the enzyme chlorophyllase. This enzyme activity causes the banana skin to turn yellow. In advanced stages of ripening, another enzyme, polyphenol oxidase, promotes the oxidation of phenols to quinones, in which the polymerization to macromolecules is then responsible for the brown pigmentation [16,24]. As can be seen from the figure, color change occurred more evenly on uncoated bananas than in coated fruit, where the surface showed brown spots on a yellow background during storage. NC samples could be considered overripe after 7 days of storage, due to the very large extension of the brown surface. A delay in the natural browning was observed in the coated bananas. When the coating was applied in combination with CaCl_2_ (HC), the fruit could actually be considered as acceptable for a longer period of time. Han and co-workers [21], who used sodium alginate/carboxymethylcellulose emulsions with cinnamon essential oil for coating bananas, observed a similar positive effect over a 7-day period when the essential oil was used at a low concentration, but higher than the LEO concentration used here.

The color change in the fruit peels during ripening was determined by measuring the L*, a*, and b* coordinates. The change in color of the banana peel from green to yellow during storage was accompanied by an increase in the a* and b* values of the color scale, and a decrease in the L* values [25]. However, small changes in L* were observed in all samples until day 4 (Table 1), before the gradual decreasing trend due to the changes to darker colors in the ripe fruit beginning.

Nevertheless, the decreasing trend was slower in the EC and HC samples compared to the NC samples. After 14 days, the L* value of HC was higher than that of the others. The increase in a* values may be related to the change in color of the banana peels from green (negative a* values) to dark, which corresponds to the decrease in chlorophyll concentration [15]. On the fourth day of storage, the NC sample already showed positive a* values (corresponding to the red color), while the EC and HC samples still showed negative a* values, with the latter sample showing only a very slight change in the a* parameter compared to the beginning of the storage period. After 14 days, the HC sample still showed negative a* values, while the EC and NC samples showed higher similar values. 

**Figure 4:**
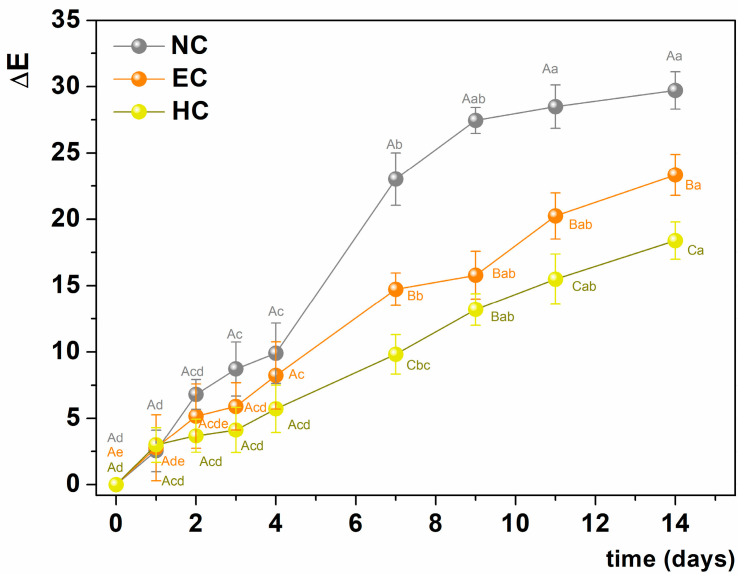
Color change (ΔE) in NC (grey), EC (orange), and HC (yellow) samples as a function of the storage time. Different uppercase letters indicate significant difference among samples (*p*-value < 0.05) in the same day; different lowercase letters indicate significant differences for the same sample over time. NC = uncoated samples; EC = samples coated with alginate emulsion; HC = samples coated with hydrogel.

The b* values, which correspond to the yellow color, increased similarly for all samples during storage. A global indicator for the evaluation of color change during ripening is ΔE, which is calculated using Equation (1). The ΔE values increased during storage. From day 2 onwards, the change in the overall peel color of uncoated bananas was twice as high in the uncoated bananas as in those coated with the hydrogel (Figure 4), demonstrating the significant effect of the coating on delaying the ripening of the bananas. The use of the emulsion without the addition of the crosslinking agent (EC) was also effective, but the effectiveness was lower than in the HC samples.

**Figure 5:**
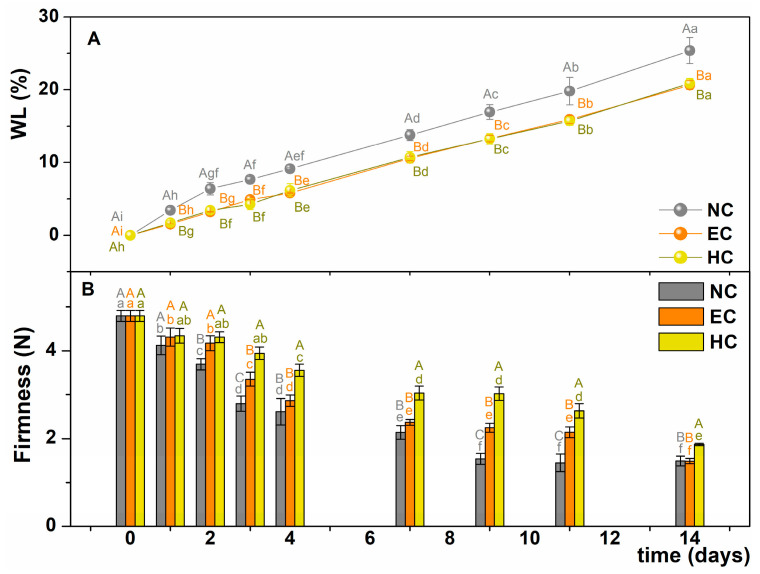
(**A**) Weight loss % (WL) and (**B**) firmness of NC (grey), EC (orange), and HC (yellow) samples as a function of the storage time. Different uppercase letters indicate significant difference among samples (*p*-value < 0.05) in the same day; different lowercase letters indicate significant differences for the same sample over time. Standard deviations are reported as error bars. NC = uncoated samples; EC = samples coated with alginate emulsion; HC = samples coated with hydrogel.

After harvest, fruits are very susceptible to weight loss (mainly due to water loss), which is accompanied by loss of freshness. As can be seen in Figure 5A, the natural moisture loss of the coated bananas was significantly slowed down thanks to the edible coating. In fact, a difference of about 3–4% in percentage weight loss (WL) was observed between the control sample and the coated samples throughout the storage period.

However, the crosslinking agent did not appear to affect the WL values. A slower increase in WL was also observed by Thakur et al. [18] and Yang et al., who coated bananas with a mixture of rice starch and carrageenan with sucrose ester and an emulsion of sodium alginate and tea tree essential oil with TiO_2_ nanoparticles, respectively. In other cases, coating with chitosan did not lead to a significant delay in weight loss [1]. Therefore, the chemical nature of the polysaccharides or their combination with other molecules may produce different permeability properties.

Another parameter subject to important changes during ripening is the firmness of the peel and pulp of the banana. Figure 5B illustrates the changes in firmness measured on the pulp of coated (EC and HC) and uncoated (NC) banana samples. The graph shows a general decrease in firmness, which is an expected phenomenon during fruit ripening.

The decrease in firmness is related to the degradation of starch into sugar and the migration of water from the peel to the pulp. These two processes change the cell stiffness, making the pulp softer [26]. As can be seen in Figure 5B, the firmness decreased more slowly with HC than with EC and more quickly with NC. There are significant differences between the samples up to the 11th day of storage, suggesting that minimizing the penetration of gas onto the banana surface effectively delays the softening of the pulp. These results are consistent with those of other authors who have studied the changes in pulp hardness to evaluate the effectiveness of coatings with modified starch, cellulose, and chitosan [13], alone and in combination with gum arabic [16].

**Figure 6:**
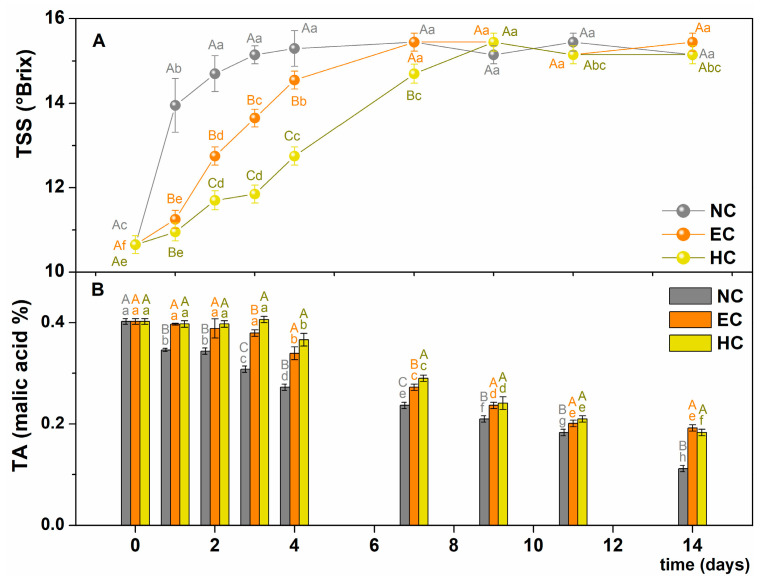
(**A**) Total soluble solid (TSS) and (**B**) titratable acidity (TA) of NC (grey), EC (orange), and HC (yellow) samples as a function of the storage time. Different uppercase letters indicate significant difference among samples (*p*-value < 0.05) in the same day; different lowercase letters indicate significant differences for the same sample over time. Standard deviations are reported as error bars. NC = uncoated samples; EC = samples coated with alginate emulsion; HC = samples coated with hydrogel.

The ripening of bananas leads to a shift in metabolism from starch synthesis to its breakdown into soluble sugars. The total soluble solid (TSS) mainly represents the concentration of soluble sugars [17], therefore it is expected to increase during fruit ripening. As can be seen in Figure 6A, the TSS increased rapidly in the control samples, reaching a maximum value of about 15 °Brix after 2 days of storage. In contrast, a slow and gradual increase was observed in the coated bananas, which lasted until day 7 for the EC fruit and day 9 for the HC bananas, until the same plateau was reached. The delay in TSS increase has been explained by other authors [5,13,14,15,17,20] who used edible polysaccharide-based films for coating banana peels. This effect was attributed to the change in the internal atmosphere caused by the coatings, which reduced the oxygen and/or carbon dioxide concentration and suppressed ethylene evolution. In the case of the EC and HC used here, the presence of the hydrogel appears to have contributed more to the slowing of fruit respiration than the non-crosslinked alginate emulsion.

The organic acids content is an indicator of the fruit freshness, as these acids are consumed during the respiration process [16,27]. However, the decrease in the titratable acidity (TA), calculated according to Equation (2) as % of malic acid [9], was different in the untreated bananas than in the coated fruit (Figure 6B). In fact, TA values decreased continuously from ~0.40 to ~0.10 in the NC samples, while in the coated bananas the decrease in TA started after 3 days of storage and eventually reached TA values of about 0.20% (twice the value of the NC fruit). The changes in the percentage of malic acid thus appeared to be significantly delayed by the coating of the banana surfaces, which is consistent with the delay in respiration rate. Other authors evaluated the effectiveness of polysaccharide coatings in delaying the degradation of organic acids [1,14,15,16]. However, the utilization of EC or HC both had a positive effect on ripeness.

**Figure 7:**
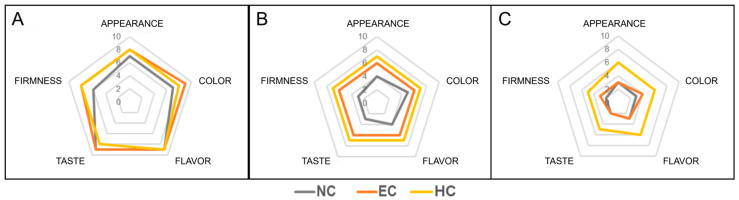
Sensory analysis of NC (grey), EC (orange), and HC (yellow) samples at (**A**) day 3, (**B**) day 7, and (**C**) day 11 of storage. NC = uncoated samples; EC = samples coated with alginate emulsion; HC = samples coated with hydrogel.

Sensory analysis of the coated or uncoated samples was performed until day 11, as the NC and EC samples were no longer suitable for such analyses after this day. The results of the sensory analysis were summarized in the form of radar plots (Figure 7A–C) comparing the scores assigned to the different attributes for the different treated samples after 3, 7, and 11 days of storage.

Figure 7A shows that on the third day of storage, the three samples were rated similarly in terms of taste and flavor, but the firmness, color, and appearance were higher for the coated samples. On the seventh day of storage (Figure 7B), the differences among the samples were perceived to be significant for all properties assessed by the judges. The NC sample was the least satisfactory of all, especially in terms of firmness and the taste. The HC sample, on the other hand, was the one rated as better. On day 11 (Figure 7C), the NC and EC samples were perceived as similar by the evaluators, with both samples receiving similarly low scores, while HC bananas were still well judged.

Table 2 shows the correlation among the investigated parameters.

All the indices used to compare the ripening process in coated or uncoated bananas significantly correlated with each other. Considering the parameter related to the peel color ΔE, it directly correlated to the WL, and TSS, indeed, as the color changed during ripening the WL and the formation of soluble sugars increased proportionally. In contrast, there was an inverse correlation between ΔE and TA, as the increase in color change corresponded to a decrease in TA, and the same was true for ΔE and firmness, which also decreased during ripening. WL with TA and firmness were also inversely correlated, while the former correlated directly with TSS. From this analysis, it can be deduced that controlling the peel color of bananas, e.g., by using suitable edible coatings, corresponds to controlling the ripening status of the fruit.

Overall, the application of an alginate emulsion crosslinked with CaCl_2_ has allowed for the extension of the shelf life of bananas by up to 11 days, which represents a contribution in this area. Looking at the results of other authors who used formulations containing alginate and/or essential oils as banana coating [19,20,21], some improvements can be outlined. In fact, despite the use of another polymer (carboxymethyl cellulose) together with alginate and cinnamon essential oil, Han et al. [21] extended the shelf life of bananas up to 7 days only; Yang et al. [20] achieved better results using an alginate emulsion with tea tree essential oil and TiO_2_ (the use of which is no longer permitted in Europe [22]), and the use of the formulation made of alginate crosslinked with whey protein isolate, very recently proposed by Deng et al. [19], has provided an improvement in shelf life of fruit by up to 8 days.

## 3. Conclusions

The use of the tested emulsion-based system with 1% sodium alginate, 1% Tween 80, and 0.1% lemongrass essential oil was found to be effective in extending the post-harvest shelf life of bananas. Significant reduction in color change, weight loss, increase in total soluble solids, decrease in titratable acidity, and slower softening of the pulp was observed in the coated bananas. It was also found that all analyzed parameters correlated strongly with each other. The effectiveness of the coating was increased by using a 2% calcium chloride solution as a crosslinking agent for alginate emulsion, which extended the shelf life by up to more than 11 days, taking into account the results of the physico-chemical parameters and the sensory analysis. This is an important aspect to consider in the food industry, as the use of the proposed coating formulation can help to reduce the production of waste from both an economic and environmental point of view, thus saving significant costs.

## 4. Materials and Methods

### 4.1. Materials

Fresh bananas (cv. Cavendish) at the physiological mature green stage and free of visible physical and fungal infection, and lemongrass (*Cymbopogon nardus*) essential oil (LEO) (Erbamea, San Giustino, Perugia, Italy) were purchased from local shops. Food-grade sodium alginate was purchased from Farmalabor (Canosa Di Puglia, Italy), while Tween 80 (Polysorbate 80), calcium chloride (CaCl_2_), sodium hydroxide (NaOH) were purchased from Merck (Merck, Darmstadt, Germany).

### 4.2. Edible Coating Formulation

To prepare the emulsion, sodium alginate (1% *w*/*w*) was dissolved in water under continuous stirring at 70 °C using a water bath. The coarse primary dispersion was made by mixing aqueous sodium alginate suspension, LEO (0.5% *w*/*w*) as lipidic phase. and Tween 80 (5% *w*/*w*) with a laboratory T25 digital Ultra-Turrax mixer (IKA, Staufen, Germany) with an S25N-8G probe, working at 24.000 rpm for 4 min. The dispersion was then subjected to ultrasonic treatment (Ultrasonic Homogenizer Model 300 VT, BioLogics Inc., Manassas, VA, USA) for 1 min at 120 W with 50% pulsed frequency to achieve finely dispersed hydrophobic particles providing stable suspensions [7]. The obtained dispersion was then diluted with the 1% alginate to achieve final LEO and Tween 80 concentrations of 0.1 and 1% *w*/*w*, respectively, and homogenized at 18.000 rpm for 2 min with an S25N-18 G probe. The final emulsion was applied to the banana’s peel using a laboratory airbrush (Model 350-9 Badger Ari-Brush Co., Bellwood, IL, USA). A 2% calcium chloride solution (*w*/*v*) was then applied in the same way as the crosslinking agent to promote the formation of the hydrogel film constituting the final coating [28]. All samples were air-treated for 1 min until complete film formation to study the effects of the coating on the development of various parameters (vide infra) during storage.

### 4.3. Edible Coating Characterization

The surface tension difference of alginate suspension and emulsion were measured at 20 °C with a digital tensiometer (DCA, digital tensiometer, Gibertini, Elettronica srl., Novate Milanese, Milano, Italy) using the Wilhelmy plate device. Edible coatings were characterized for rheological response to oscillatory mechanical tests using a modular rheometer (Haake MARS III-Thermo Scientific, Karlsruhe, Germany) equipped with a 60 mm parallel plate geometry probe at a gap distance of 0.07 mm to mimic the thickness of the coating applied on the banana fruit surface. The temperature, controlled by a cooling and heating system (Phoenix II, Thermo Scientific, Karlsruhe, Germany), combined with a Peltier system, was set at 20 °C. For studying the behavior of the coating made of alginate emulsion, the dispersion was sprayed with the airbrush on the lower plate, and for crosslinked hydrogel a second step of CaCl_2_ spraying followed. Amplitude sweep tests were carried out in control stress mode by varying the applied stress according to the sample differences, and keeping the frequency fixed at 1 Hz. Frequency sweep tests were made in a frequency range between 0.1 and 10 Hz and by applying a stress taken from the linear viscoelastic region. The stress applied for alginate emulsion and for crosslinked hydrogel were of 0.05 and 0.01 Pa, respectively.

### 4.4. Shelf Life of Coated Fruit

Color of the banana peel was taken by a colorimeter (Minolta CR-300, Minolta Italia S.p.A., Milan, Italy) using the CIE Lab color space. Since the peel color was not consistent over the entire surface area, measurements were taken on the apical, middle, and basal part of fruit. Mean values of 6 measurements were calculated. The color values of L*, a*, and b* refer to lightness, redness, and yellowness, respectively. The color difference (Δ*E*) was quantified at each time through Equation (1):

(1) $$\Delta E=\sqrt{{\left(\Delta L^{*}\right)}^{2}+{\left(\Delta a^{*}\right)}^{2}+{\left(\Delta b^{*}\right)}^{2}}$$

where ΔL*, Δa*, and Δb* represent the variation of L*, a*, and b* values from the beginning of the storage.

The weight loss percentage of bananas was calculated by considering the differences between the initial weight and the weight during storage of the whole banana fruit, to the initial weight multiplied by 100.

Total soluble solids (TSSs) were determined through a refractometer (Atago N-1E, Tokyo, Japan). Samples were prepared according to Lustriane et al. [17] with slight modifications. A total of 15 g of pulp was homogenized for 30 s with 45 mL of distilled water. The mixture was centrifuged at 9000 rpm for 10 min at 20 °C and one drop of supernatant was placed on the prism of the refractometer, and results were expressed as Brix degree (°Brix). Analysis was carried out in triplicate analyzing three randomly selected samples for each replicate.

For determination of titratable acidity (*TA*), samples were prepared as reported above. Titratable acidity (*TA*) was determined through titration with NaOH 0.1 N until pH 8.2 was reached. Values were expressed as percentage malic acid according to the following Equation (2) [29]:

(2) $$TA\left(\%\;malic\;acid\right)=\frac{V\;N\;Eq_{wt}}{W\;1000}\;100$$

where *V* and *N* are the mL of NaOH used for the titration and the concentration (0.1 N), *Eq_wt_* is the equivalent weight of malic acid, and *W* is the sample weight. Analysis was carried out in triplicate analyzing three randomly selected samples for each replicate.

The textural characteristics of bananas during storage were measured through a texture analyzer (TA.XT2, Stable Micro System, Godalming, UK) using a 5 kg load cell and a 6 mm cylindrical probe (P/6) to assess fruit hardness through the penetration test. The following settings were used: pre-test speed = 1.5 mm/s, test speed = 1.0 mm/s, post-test speed = 10.0 mm/s, distance = 50%. The peak force to penetrate the sample, recorded on a force–time curve, was taken as the hardness [25]. The mean of the measurements made on seven peeled samples was considered for each type of sample during the storage.

### 4.5. Sensory Analysis

The sensory evaluation of the samples for overall acceptability, pulp color, taste, flavor, and texture was performed by 10 untrained panelists (aged between 25 and 55 years; equally divided among males and females) based on the method of Maqbool et al. [30]. Samples coded with a 3-digit code were randomly served to the panelists which were asked to score the intensity of the selected attributes (appearance, color, taste, flavor, and firmness) according to a hedonic scale of 9 points, where 0–2 represented extreme dislike; 3–5, fair; 6–8, good; and 9, excellent. All subjects involved in the sensory study were preliminary informed about the nature of the research and agreed to participate. No personal data were collected or used in any form.

### 4.6. Statistical Analysis

SPSS software (version 22.0, IBM Statistics, Armonk, NY, USA) was used for the statistical analysis. Data were evaluated by the analysis of variance with multiple comparisons (MANOVA) using the Tukey post-hoc test to determine differences among the means. Within the plots, different uppercase letters indicate significant difference among the samples (*p*-value < 0.05) at each time, while different lowercase letters indicate significant differences for each sample over time. Standard deviations are reported as error bars. Bivariate correlations among the parameters were checked through Pearson’s correlation index considering the minimum levels of significance of *p*-value < 0.05.

## Figures and Tables

**Table 1:** L*, a*, b* indices of NC, EC, and HC samples during the storage time. Different uppercase letters indicate significant difference among samples (*p*-value < 0.05) in the same day; different lowercase letters indicate significant differences for the same sample over time.

	L*			a*			b*		
Days	NC	EC	HC	NC	EC	HC	NC	EC	HC
0	72.35 ± 1.20 Ab	71.14 ± 1.33 Aa	73.42 ± 1.43 Aa	−5.42 ± 0.97 Ad	−5.02 ± 0.84 Ae	−5.93 ± 0.73 Ad	35.76 ± 1.67 Ac	36.46 ± 1.41 Ade	33.14 ± 1.32 Bf
1	71.51 ± 0.72 Ab	70.16 ± 1.36 Aa	71.37 ± 1.05 Aa	−5.90 ± 0.48 Ad	−6.17 ± 0.48 Af	−6.60 ± 0.44 Ae	35.65 ± 1.45 Ac	35.12 ± 1.18 Ae	34.89 ± 1.71 Af
2	72.33 ± 1.53 Ab	72.01 ± 1.63 Aa	71.33 ± 0.58 Aa	−1.65 ± 0.14 Ac	−3.89 ± 0.41 Bd	−6.48 ± 0.42 Ce	36.81 ± 1.42 ABc	34.67 ± 1.04 Be	37.95 ± 1.76 Ae
3	72.65 ± 1.11 Ab	69.84 ± 1.01 Ba	71.43 ± 0.80 Aa	0.49 ± 0.34 Aa	−1.28 ± 0.34 Bc	−6.41 ± 0.24 Ce	41.64 ± 1.19 Ab	37.26 ± 1.24 Bd	39.39 ± 1.10 ABde
4	75.30 ± 0.93 Aa	71.47 ± 1.06 Ba	72.47 ± 1.10 Ba	1.22 ± 0.12 Aa	−1.04 ± 0.03 Bc	−5.48 ± 0.25 Cd	42.12 ± 1.03 Ab	42.58 ± 1.06 Ac	41.16 ± 1.19 Acd
7	65.98 ± 2.27 Ac	61.96 ± 1.44 Bb	64.89 ± 0.13 Ab	1.05 ± 0.65 Aa	0.21 ± 0.34 Aab	−2.72 ± 0.31 Bc	50.40 ± 2.46 Aa	44.07 ± 2.24 Bbc	42.27 ± 1.23 Bc
9	57.61 ± 0.86 Cd	63.67 ± 1.21 Bb	67.29 ± 1.85 Ab	1.19 ± 0.30 Aa	−0.27 ± 0.28 Bb	−1.74 ± 0.24 Cb	50.44 ± 1.35 Aa	47.64 ± 1.16 Bb	47.00 ± 1.70 Bb
11	55.61 ± 0.14 Ce	60.33 ± 0.62 Bb	64.96 ± 1.71 Ab	1.45 ± 0.73 Aa	0.91 ± 0.40 Aa	−0.77 ± 0.24 Ba	50.28 ± 1.37 Aa	50.43 ± 1.21 Aa	48.43 ± 1.46 Aab
14	53.28 ± 0.66 Cf	55.67 ± 0.92 Bc	62.62 ± 1.51 Ab	1.30 ± 0.77 Aa	0.89 ± 0.39 Aa	−0.61 ± 0.49 Ba	50.89 ± 1.42 Aa	50.60 ± 1.89 Aa	50.43 ± 1.24 Aa

NC = uncoated samples; EC = samples coated with alginate emulsion; HC = samples coated with hydrogel.

**Table 2:** Pearson correlation coefficients among the investigated parameters.

	WL	TA	TSS	Firmness
ΔE	0.931 **	−0.924 **	0.755 *	−0.923 *
WL		−0.976 **	0.796 *	−0.934 *
TA			−0.821 *	0.928 *
TSS				−0.870 **

* correlation is significant at the 0.05 level; ** correlation is significant at the 0.01 level.

## Data Availability

The raw data supporting the conclusions of this article will be made available by the authors on request.
